# Investigation of serum β-defensin-1 level in calves with coccidiosis

**DOI:** 10.5455/javar.2021.h539

**Published:** 2021-09-20

**Authors:** Akın Koçhan

**Affiliations:** Faculty of Veterinary Internal Medicine, Dicle University, Diyarbakir, Turkey

**Keywords:** β-defensin-1, calf, coccidiosis, level, serum

## Abstract

**Objective:**

Coccidiosis is a protozoan infection that can result in hemorrhagic diarrhea, depression, weakness, weight loss, and even mortality in young animals. β-defensin-1 is an antimicrobial peptide produced largely by epithelial cells in the skin and mucosa. It possesses antifungal, antibacterial, antiparasitic, and antiviral properties. The goal of this study was to evaluate how β-defensin-1 levels changed in coccidiosis-infected calves.

**Materials and Methods:**

The sample included 10 coccidiosis-positive calves and 7 healthy calves, for a total of 17 calves of diverse breeds and older than 15 days. To assess the level of β-defensin-1, blood samples were obtained from the vena jugularis of the animals. The concentrations of β-defensin-1 in the serum were measured using a commercial ELISA kit.

**Results:**

Although the serum β-defensin-1 level decreased in infected animals, the drop was not statistically significant when compared to the control group.

**Conclusion:**

According to the study’s findings, there was no significant change in the serum β-defensin-1 level in coccidiosis-infected calves. We believe that it will be advantageous to conduct additional studies with a larger sample size in order to acquire more precise results.

## Introduction

Coccidiosis is a protozoan disease that can result in hemorrhagic diarrhea, depression, weakening, weight loss, and sometimes death, especially in young animals caused by *Eimeria* (*E.*) *zuernii*, *Eimeria bovis*, and *Eimeria aubornensis* species of the *Eimeriidae* family [1,2]. Coccidiosis is prevalent throughout the world, affecting primarily poultry and animals such as cattle, sheep, goats, dogs, cats, pigs, and rabbits [1,3].

Death, poor performance, increased susceptibility to other diseases, prevention and treatment costs are important economic losses due to coccidiosis, especially in breeding farms and calf breeding systems [1]. Infection occurs through taking sporulated *Eimeria* oocysts orally from water or feed [3,4]. By disintegrating the intestinal epithelium, Coccidia pathogens cause epithelial cell shedding on the mucosal surfaces, the uncovering of lamina propria, and the atrophy in the intestinal villus [5]. As a result, intestinal bleeding, diarrhea, fluid-electrolyte loss, hypoproteinemia, anemia, acidosis, shock, and secondary bacterial infections may result in death [6].

Antimicrobial peptides are a class of small peptides that consist of less than 100 amino acids, and they are an essential part of the innate immune system of different organisms [6,7]. In mammals, there are three crucial antimicrobial peptide groups: cathelicidins, histatins, and defensins. An essential antimicrobial peptide group in mammals is defensins [6]. Defensins are arginine-rich cationic molecules consisting of 30–40 amino acids, with molecular weights of 3.5–6 kDa, and containing three disulfide bridges constituted by six cysteine residues [6,8]. Based on the cysteine residues and the location of the disulfide bonds, they are divided into three subgroups as alpha (α), beta (β), and theta (θ) defensins [6–10]. Primary epithelial cells and neutrophils synthesize β-defensins serially with low energy, and they can be easily stored in large quantities [6,9]. β-defensins are resting ready where they are stored. Their levels rise rapidly following infection. They rapidly inhibit the growth of various microorganisms, forming synergistic interactions with one another or with other components of the natural host defense system, such as lysozyme and lactoferrin as with various antibiotics [7,10]. β-defensins secreted from more than one tissue in cattle are called bovine neutrophil β-defensin (BNBD) [11]. Antimicrobial activities of bovine β-defensins against pathogens such as *Staphylococcus aureus*, *Escherichia coli*, *Pseudomonas aeruginosa*, *Klebsiella pneumoniae*, and *Candida* spp*.* have been reported [12]. It is stated that alongside their antifungal, antibacterial, antiparasitic, and antiviral activities, β-defensins can contribute to epithelial proliferation and differentiation during wound healing [13].

β-defensin-1 is an antimicrobial peptide that is primarily synthesized by epithelial cells in the skin and mucosa and exhibits antifungal, antibacterial, antiparasitic, and antiviral activity. The purpose of this study was to determine the changes in β-defensin-1 levels in calves with coccidiosis.

## Material and Method

The material of the study consisted of calves older than 15 days old brought to Dicle University Veterinary Faculty with the complaint of diarrhea. The control group consisted of seven healthy calves. Stool samples were taken from the rectum of calves with diarrhea into sterile stool containers. We studied rotavirus, coronavirus, cryptosporidium, *E. coli* F5 (K99), and *Clostridium perfringens* pathogens using a rapid diagnostic kit (Rainbow Calf Scours -BIO K 306 Ag Test Kit, Biox Diagnostics, Belgium). Ten calves with coccidiosis in which these pathogens were not detected were included in the study. The control group consisted of seven healthy calves. The samples were examined using simple stool examination techniques simple and flotation techniques to determine the *Eimeria* oocysts. A standard and improved Modified McMaster method was used to detect and enumerate coccidial oocysts.

Blood samples of diseased animals were taken from vena jugularis into tubes with and without EDTA using a sterile cannula. Blood samples were analyzed hematologically using a Mindray BC-2800 Vet blood count device without waiting for coccidiosis screening, and those found to have coccidiosis were included in this study. After centrifuging samples for 10 min at 3,000 × gm, they were stored at −20°C until analysis, and serums from animals positive for coccidiosis were examined biochemically using a Fujifilm DRI-CHEM NX500 brand biochemistry apparatus. Serum β-defensin-1 level was determined using the commercial ELISA kit (SinoGeneClon Biotech Co., Ltd).

The computerized data program IBM Statistical Package for the Social Sciences Statistics for Windows, version 21 (IBM Corp., Armonk, NY) was used to analyze the data. The difference between the groups was compared using Student’s *t*-test. The level of *p* < 0.05 was considered to be statistically significant.

## Results and Discussion

A decrease in β-defensin-1 level was observed in infected animals, but it was not statistically significant (*p* > 0.05) compared to the control group (Table 1). 

The hematological examination revealed a statistically significant rise in the number of white blood cell (WBC), monocytes (Mon), hemoglobin (HGB), hematocrit level (HTC), platelet distribution width (PDW), and red blood cell (RBC) (*p* < 0.05) and a drop in the level of mean corpuscular haemoglobin (MCH) (*p* < 0.05) when compared to the control group (Table 2). In biochemical analysis, the decrease in the levels of alkaline phosphatase (ALP), phosphorus (P), sodium (Na) (*p* < 0.005), glucose (GLU), calcium (Ca), magnesium (Mg), (*p* < 0.05), and the increase in the level of total bilirubin (TBIL) (*p* < 0.05) were found statistically significant compared to the control group (Table 3).

Coccidia pathogens cause intestinal bleeding, diarrhea, fluid-electrolyte loss, hypoproteinemia, anemia, acidosis, shock, and secondary bacterial infections by disintegrating the intestinal epithelium and causing epithelial cell shedding on mucosal surfaces, uncovering of the lamina propria, and villus atrophy [1,5]. Antimicrobial peptides (AMPs) are generated serially at low energy by epithelial and phagocytic cells, particularly on mucosal surfaces, and are easily stored in vast amounts. These peptides, which are readily available where they are stored, rapidly accumulate in high concentrations following infection and inhibit the growth of a variety of microorganisms via synergistic interactions with one another or with other natural host defense elements such as lysozyme and lactoferrin. It is also reported that they have a synergistic effect with various antibiotics. Additionally, β-defensins have been shown to promote epithelial proliferation and differentiation during wound healing [7,13,14]. Wei et al. [15] reported that different types of β-defensins or lysozymes might work together to defend against potential pathogens.

Increased serum β-defensin levels have been observed in patients with lung cancer [16], diffuse panbronchiolitis [17], and cirrhosis [18]. Jaradat et al. [19] reported an increase in serum β-defensin-2 levels in patients infected with *Trichophyton rubrum*. In contrast, Tsybikov et al. [20] reported that plasma alpha defensin levels increased during the exacerbation period in patients with atopic dermatitis, with a positive correlation between the clinical severity of the disease and the alpha defensin level.

Veldhuizen et al. [21] shown that infection with *Salmonella typhimurium* did not alter the expression of β-defensin-1 and β-defensin-2 in the intestines of pigs. Wang et al. [22] found that the concentration of BNBD-1 in colostrum on the day following calving was higher than on the 5th day.

**Table 1.** Serum β-defensin-1 levels of the calves with coccidiosis and those in the control group.

Parameter	*N*	Maximum	Minimum	Mean	Standard deviation	*p*-value
β-defensin-1 (ng/ml)	Control 7	2.542	0.738	1.627	0.675	0.117
	İnfected10	1.938	0.234	1.088	0.645	

**Table 2.** Hematological findings of the calves with coccidiosis and of the control group.

Parameters	*N*	Maximum	Minimum	Mean	Standard deviation	*p*-value
WBC (10^9^/l)	Control 7	8.90	5.00	7.00	1.23	0.020*
	İnfected 10	10.50	7.2	8.69	1.36	
Lymph (10^9^/l)	Control 7	4.60	2.10	3.51	0.91	0.214
	İnfected 10	5.20	3.00	4.05	0.78	
Mon (10^9^/l)	Control 7	1.00	0.70	0.85	0.12	0.006*
	İnfected 10	1.30	0.80	1.11	0.17	
Granulocytes (Gran) (10^9^/l)	Control 7	3.80	1.90	2.62	0.65	0.057
	İnfected 10	4.90	2.00	3.53	1.00	
Lymph (%)	Control 7	61.00	42.00	49.58	7.02	0.433
	İnfected 10	58.60	37.40	46.66	7.57	
Mon (%)	Control 7	15.40	11.10	12.68	1.63	0.675
	İnfected 10	16.80	8.80	13.10	2.16	
Gran (%)	Control 7	43.60	26.20	37.78	6.96	0.489
	İnfected 10	50.20	26.90	40.24	7.32	
RBC (10^9^/l)	Control 7	8.95	7.55	8.30	0.56	0.001**
	İnfected 10	11.49	7.86	10.30	1.16	
HGB (g/dl)	Control 7	11.00	8.70	9.67	0.89	0.008*
	İnfected 10	12.20	9.10	11.17	1.06	
HCT (%)	Control 7	33.30	25.70	28.90	2.71	0.009*
	İnfected 10	37.80	26.80	33.81	3.64	
MCV (fl)	Control 7	38.30	32.60	34.81	1.96	0.237
	İnfected 10	39.90	32.00	33.48	0.15	
MCH (pg)	Control 7	12.60	11.00	11.58	0.55	0.009*
	İnfected 10	11.70	10.20	10.84	0.46	
MCHC (gm/dl)	Control 7	34.10	32.70	33.42	0.51	0.576
	İnfected 10	35.60	30.20	33.13	1.52	
RDW (%)	Control 7	18.90	15.80	17.57	1.20	0.079
	İnfected 10	23.40	16.20	19.35	2.26	
PLT (10^9^/l)	Control 7	634.00	313.00	494.43	133.08	0.174
	İnfected 10	751.00	460.00	574.20	97.95	
Mean platelet volume (fl)	Control 7	4.50	3.60	3.95	0.28	0.150
	İnfected 10	4.70	3.70	4.17	0.28	
PDW	Control 7	16.10	15.70	15.87	0.12	0.042*
	İnfected 10	16.40	15.80	16.07	0.21	
Procalsitonin (%)	Control 7	0.26	0.12	0.19	0.05	0.072
	İnfected 10	0.31	0.17	0.23	0.04	

** *p* < 0.005,

* *p* < 0.05 in comparison with control group.

**Table 3.** Serum biochemical findings of the calves with coccidiosis and those in the control group.

Parameters	*N*	Maximum	Minimum	Mean	Standard deviation	*p*-value
ALP (UI/l)	Control 7	270	138	188.86	47.32	0.003**
	İnfected 10	180	53	118.2	33.4	
Glutamate oxaloacetate transaminase (UI/l)	Control 7	67	51	60	6.05	0.301
	İnfected 10	145	40	71.80	28.42	
GGT (UI/l)	Control 7	50	14	23.57	12.05	0.089
	İnfected 10	20	11	16.5	2.63	
Glutamate pyruvate transaminase (UI/l)	Control 7	31	11	18.57	7.09	0.478
	İnfected 10	27	15	20.5	3.83	
Creatine kinase (UI/l)	Control 7	129	82	110.14	17.46	0.109
	İnfected 10	478	74	185.5	115.0	
Creatine kinase- myocard base (UI/l)	Control 7	300	91	153.14	73.05	0.232
	İnfected 10	300	112	195.3	65.56	
BUN (mg/dl)	Control 7	10.2	6.00	8.8	1.44	0.083
	İnfected 10	19.7	5.00	12.05	5.10	
Creatine (mg/dl)	Control 7	1.2	0.7	0.92	0.17	0.862
	İnfected 10	1.5	0.7	0.91	0.23	
TP (UI/l)	Control 7	6.8	5.4	5.92	0.47	0.679
	İnfected 10	8.2	4.7	6.1	0.98	
ALB (gm/dl)	Control 7	3.30	2.80	3.15	0.18	0.018*
	İnfected 10	3.40	2.20	2.80	0.31	
TBIL (mg/dl)	Control 7	030	0.20	0.22	0.04	0.031*
	İnfected 10	070	0.20	0.36	0.15	
GLU (mg/dl)	Control 7	109	69	84.57	17.26	0.118
	İnfected 10	125	40	65.4	26.79	
Ca (mg/dl)	Control 7	11.10	9.80	10.47	0.41	0.020*
	İnfected 10	10.80	8.10	9.62	0.78	
Mg (mg/dl)	Control 7	2.80	2.10	2.31	0.24	0.012*
	İnfected 10	2.30	1.80	2.05	0.13	
P (mg/dl)	Control 7	11	9.50	10.35	0.55	0.001**
	İnfected 10	10	6	8.31	1.30	
Na (mEq/l)	Control 7	143	136	140	2.64	0.004**
	İnfected 10	139	127	134.1	3.92	
K (mEq/l)	Control 7	5.30	4.20	4.75	0.475	0.226
	İnfected 10	5.50	3.80	4.45	0.506	
Cl (mEq/l)	Control 7	108	101	104.29	2.69	0.513
	İnfected 10	132	85	101.1	12.25	

** *p* < 0.005,

* *p* < 0.05 in comparison with control group.

Elahi et al. [23] report that *in vivo* treatment with porcine β-defensin-1 protects newborn piglets against *Bordetella pertussis* respiratory infection and that AMPs may be used in place of antibiotics. According to Ramasundara et al. [24] and Fellermann et al. [25], AMPs exert antibacterial activity in the gastrointestinal tract by producing microporosity in the phospholipid layer of bacterial membranes, resulting in the loss of structural integrity and cell collapse. According to Salzman et al. [26], defensins protect the host epithelium and stem cells from aggressive infections and also contribute to the regulation of the commensal microbiota’s number and composition.

According to Ackermann et al. [27], the decreased sheep β-defensin-1 expression observed during acute *Mannheimia haemolytica* pneumonia is in stark contrast to the enhanced sheep β-defensin-1 expression shown during parainfluenza virus type 3 (PI-3) infection. According to Jin et al. [28], *Saccharomyces cerevisiae* mannan induces sheep β-defensin-1 in a concentration- and time-dependent way and that the effect is diminished when the mannan concentration is too low or too high.

Tarver et al. [29] observed that pathogens such as *Cryptosporudium parvum*, crypt cells may contribute to immunization for defense against these microorganisms by releasing antimicrobial peptides that produce histological alterations in the intestines. Additionally, they reported that a novel member of the β-defensin family of antimicrobial peptides is inducibly expressed in enteric epithelial cells of the colon and distal small intestine, enabling enteric epithelial cells to participate dynamically in local host defense or enteric flora regulation. According to Friend and Stockdale [5], coccidia pathogens, such as *C. parvum*, produce histological alterations (e.g., epithelial cell shedding in the mucosal surfaces through the disintegration of the intestinal epithelium, crypt-cell hyperplasia). 

It is well established that diarrhea alters hematological and biochemical markers. According to Nafie et al. [30], diarrhea episodes resulting in moderate dehydration resulted in a drop in RBC and hematocrit values and an increase in WBC, hemoglobin (Hb), mean corpuscular volume (MCV), MCH, and mean corpuscular hemoglobin concentration (MCHC) values when compared to the control group. Ozkan and Akgul [31] reported that they observed an increase in WBC, hematocrit, and HGB values in moderately dehydrated calves with diarrhea. Sarı and Onmaz [32] found that dogs with giardiasis had increased WBC, hematocrit, Hb, MCHC, and PLT (platelet) values when compared to the control group.

Bangoura and Daugschies [33] found an increase in erythrocytes, HGB, MCV, MCH, and hematocrit values, as well as a decrease in red cell distribution width (RDW) and PDW values in calves infected with *E. zuernii* oocysts at 250,000 spores per calf. Yilmaz et al. [34] found a rise in RBC, hematocrit, and HGB levels and a decrease in WBC values in coccidiosis-infected beef calves compared to the control group. Eglenti et al. [35] revealed that the values of WBC, haematocrit (HCT), Mon, Neu (percent), and MCV in coccidiosis calves were significantly greater than those in the control group, although lymphocyte (Lymph) and MCHC values were substantially lower. In this study, a statistically significant rise in the amount of WBC, Mon, HGB, HCT, RDW, and RBC was seen when compared to the control group.

Nafie et al. [30] discovered a drop in serum total protein (TP), albumin (ALB), and Na levels and an increase in serum potassium (K) and chlorine (Cl) (levels in diarrhea patients). On the other hand, Ozkan and Akgul [31] observed a decrease in GLU, Na, K, and chloride and increased TP, ALB, blood urea nitrogen (BUN), and creatinine. According to Sarı and Onmaz [32], serum aspartate aminotransferase (AST), ALP, lactate dehydrogenase (LDH), and Ca levels decreased in dogs with giardiasis, while GLU, BUN, ALT, gamma glutamyl transferase (GGT), P, amylase, and lipase levels increased. Eglenti et al. [35] reported that the urea and creatinine concentrations of the diseased calves were significantly higher than those of the control group, while the TP, ALB, and globulin concentrations of the diseased calves were significantly lower than those of the control group. The present investigation discovered statistically significant decreases in the levels of ALP, P, Na, GLU, Ca, and Mg and an increase in the level of TBIL compared to the control group. We believe that the discrepancies in biochemical parameters shown in previous studies [30–32,35] are due to the animals’ age and gender, degree of dehydration, and degree of liver damage.

As a result, the current investigation sought to identify changes in serum β-defensin-1 levels that may occur due to pathological histological alterations in the intestines during the course of coccidiosis. There was a drop in serum β-defensin-1 levels in coccidiosis-infected calves, although it was not statistically significant when compared to the control group (*p* > 0.05).

## Conclusion

While serum levels of β-defensin-1 fell in infected animals, the decrease was not statistically significant when compared to the control group. This work contributes to the field of knowledge by examining the serum β-defensin-1 level in coccidiosis calves, but it would be helpful to expand the number of samples and also to evaluate the -defensin-1 level in tissue to gain more efficient results.

## List of Abbreviations

ALB; albumin, ALP; alkaline phosphatase, BUN; blood urea nitrogen, Ca; calcium, Cl; chlorine, GGT; gamma glutamyl transferase, GLU; glucose, Gran; granulocytes, HCT; haematocrit, HGB; hemoglobin, K; potassium, Lymph; lymphocytes, MCH; mean corpuscular haemoglobin, MCHC; mean corpuscular hemoglobin concentration, MCV; mean corpuscular volume, Mg; magnesium, Mon; monocytes, RBC; red blood cell, RDW; red cell distribution width, PLT; platelet, Na; sodium, P; phosphorus, PDW; platelet distribution width, TBIL; total bilirubin,TP; total protein, WBC; white blood cell.

## References

[1] Aksoy G, Gul Y, Gul Y (2012). Peritoneum ve Mesenterium Hastalıkları. Geviş Getiren Hayvanların İç Hastalıkları.

[2] Jolley WR, Bardsley KD (2006). Ruminant coccidiosis. Vet Clin North Am Food Anim.

[3] Dubey JP (2018). A review of coccidiosis in water buffaloes (*Bubalus bubalis*). Vet Parasitol.

[4] Ernst JV, Benz GW (1986). Intestinal coccidiosis in cattle. Vet Clin North Am Food Anim.

[5] Friend SC, Stockdale PH (1980). Experimental *Eimeria bovis* infection in calves: a histopathological study. Can J Comp Med.

[6] Sabanoglu E, Turutoglu H (2016). Hayvanlarda defensinler ve özellikleri. MAE Vet Fak J.

[7] Brogden KA, Ackermann M, McCray PB, Tack BF (2003). Antimicrobial peptides in animals and their role in host defences. Int J Antimicrob Agents.

[8] Gürpınar S, Kırkan Ş (2010). Antimikrobiyel Peptidler. Istanbul Univ Vet Fak Derg.

[9] De Smet K, Contreras R (2005). Human antimicrobial peptides: defensins, cathelicidins and histatins. Biotechnol Lett.

[10] Hazlett L, Wu M (2011). Defensins in innate immunity. Cell Tissue Res.

[11] Selsted ME, Tang YQ, Morris WL, McGuire, PA, Novotny MJ, Smith W (1993). Purification, primary structures, and antibacterial activities of beta-defensins, a new family of antimicrobial peptides from bovine neutrophils. J Biol Chem.

[12] Diamond, G, Beckloff N, Weinberg A, Kisich KO (2009). The roles of antimicrobial peptides in innate host defense. Curr Pharm Des.

[13] Izadpanah A, Gallo RL (2005). Antimicrobial peptides. J Am Acad Dermatol.

[14] Chanu KV, Thakuria D, Kumar S (2018). Antimicrobial peptides of buffalo and their role in host defenses. Vet World.

[15] Wei C, Tang X, Wang F, Li Y, Sun L, Luo F (2019). Molecular characterization of pulmonary defenses against bacterial invasion in allergic asthma: the role of Foxa2 in regulation of β-defensin 1. PLoS One.

[16] Arimura Y, Ashitani JI, Yanagi S, Tokojima M, Abe K, Mukae H (2004). Elevated serum β-defensins concentrations in patients with lung cancer. Anticancer Res.

[17] Hiratsuka T, Mukae H, Iiboshi H, Ashitani J, Nabeshima K, Minematsu T (2003). Increased concentrations of human β-defensins in plasma and bronchoalveolar lavage fluid of patients with diffuse panbronchiolitis. Thorax.

[18] Kaltsa G, Bamias G, Siakavellas SI, Goukos D, Karagiannakis D, Zampeli E (2016). Systemic levels of human β-defensin 1 are elevated in patients with cirrhosis. Ann Gastroenterol.

[19] Jaradat SW, Cubillos S, Krieg N, Lehmann K, Issa B, Piehler S (2015). Low DEFB4 copy number and high systemic hBD-2 and IL-22 levels are associated with dermatophytosis. J Invest Dermatol.

[20] Tsybikov NN, Petrisheva IV, Fefelova EV, Kuznik BI, Magen E (2016). Plasma α-defensins are elevated during exacerbation of atopic dermatitis. Clin Exp Dermatol.

[21] Veldhuizen EJA, Dijk A, Tersteeg MHG, Kalkhove SIC, Meulen J, Niewold TA (2017). Expression of β-defensins pBD-1 and pBD-2 along the small intestinal tract of the pig: lack of upregulation in vivo upon *Salmonella typhimurium* infection. Mol Immunol.

[22] Wang XF, Cao RM, Li J, Wu SM, Chen TX (2015). Concentration characteristics of bovine β-defensin 1 and 2 in fresh bovine milk and infant formula. Int J Dairy Technol.

[23] Elahi S, Buchanan RM, Attah-Poku S, Townsend HG, Babiuk LA, Gerdts V (2006). The host defense peptide beta-defensin 1 confers protection against *Bordetella pertussis* in newborn piglets. Infect Immun.

[24] Ramasundara M, Leach ST, Lemberg DA, Day AS (2009). Defensins and inflammation: the role of defensins in inflammatory bowel disease. J Gastroenterol Hepatol.

[25] Fellermann K, Wehkamp J, Herrlinger KR, Stange EF (2003). Crohn’s disease: a defensin deficiency syndrome. Eur J Gastroenterol Hepatol.

[26] Salzman NH, Underwood MA, Bevins CL (2007). Paneth cells, defensins, and the commensal microbiota: a hypothesis on intimate interplay at the intestinal mucosa. Semin Immunol.

[27] Ackermann MR, Gallup JM, Zabner J, Evans RB, Brockus CW, Meyerholz DK (2004). Differential expression of sheep beta-defensin-1 and-2 and interleukin 8 during acute *Mannheimia haemolytica* pneumonia. Microb Pathogen.

[28] Jin X, Zhang M, Cao GF, Yang YF (2019). *Saccharomyces cerevisiae* mannan induces sheep beta-defensin-1 expression via Dectin-2-Syk-p38 pathways in ovine ruminal epithelial cells. Vet Res.

[29] Tarver AP, Clark DP, Diamond G, Russell JP, Erdjument-Bromage H, Tempst P (1998). Enteric β-defensin: molecular cloning and characterization of a gene with inducible intestinal epithelial cell expression associated with *Cryptosporidium parvum *infection. Infect Immun.

[30] Nafie T, Ali A, Abd Elkhalik D (2015). Clinical and laboratory studies on diarrhea problem in newborn calves. Suez Canal Vet Med J.

[31] Ozkan C, Akgul Y (2004). Neonatal ishalli buzağilarda hematolojik, biyokimyasal ve elektrokardiyografik bulgular. YYU Vet Fak Derg.

[32] Sarı M, Onmaz AC (2011). Giardiosis’li köpeklerde hematolojik ve biyokimyasal göstergelerin değerlendirilmesi. Sağlık Bilimleri Dergisi.

[33] Bangoura B, Daugschies A (2007). Parasitological and clinical parameters of experimental *Eimeria zuernii* infection in calves and influence on weight gain and haemogram. Parasit Res.

[34] Yilmaz S, Issi M, Kandemir FM, Gul Y (2014). Malondialdehyde and total antioxidant levels and hematological parameters of beef cattle with coccidiosis. Van Vet J.

[35] Eglenti N, Kozat S, Denizhan V (2020). Investigation of immunoglobulin (IgE, IgA, IgG, IgM) concentrations in calves naturally infected with coccidiosis. J Istanbul Vet Sci.

